# The potential of maternal and child health service data in Australia: how lessons from the COVID‐19 pandemic can accelerate data‐informed decision making

**DOI:** 10.5694/mja2.52630

**Published:** 2025-03-16

**Authors:** Ashleigh Shipton, Meredith O'Connor, Melissa Wake, Sharon Goldfeld, Helen Lees, Catina Adams, Kristina Edvardsson, Leesa Hooker, Jatender Mohal, Rhiannon M Pilkington, Fiona K Mensah

**Affiliations:** ^1^ University of Melbourne Melbourne VIC; ^2^ Murdoch Children's Research Institute Melbourne VIC; ^3^ Royal Children's Hospital Melbourne VIC; ^4^ Municipal Association of Victoria Melbourne VIC; ^5^ La Trobe University Melbourne VIC; ^6^ University of Adelaide Adelaide SA

**Keywords:** Population health, Maternal health, Social determinants of health, COVID‐19, Child health, Child development, Datasets as topic, Health policy, Health planning, Health promotion, Health services, Public policy

To enable proactive decisions that promote a healthy start to life, our understanding of children's health and development at a population level is only as good as the data we collect and analyse.^1^ A decade ago, Olver called for improved access and capacity to link data in the Australian context, voiced in the *Medical Journal of Australia*.^2^ In the intervening years, there have been varying rates of progress across Australian states and territories. Victoria's routinely collected statewide data documenting children's health and development from pregnancy to school entry, the maternal and child health (MCH) service dataset, is the most comprehensive nationally with the highest population uptake, yet remains unlinked to key health and determinants data and longitudinal cohorts.^3^ The time to address this is now given increasing policy interest in the first 2000 days (conception to five years of age) demonstrated by the national Early Years Strategy 2024–2034 and the $14 billion rollout of Victoria's Best Start, Best Life reforms.^4^, ^5^, ^6^

Untapped child health data are an urgent public health concern with real‐world implications, notably the unmet need for timely monitoring and reporting of health and developmental outcomes of Australian children born during the coronavirus disease 2019 (COVID‐19) pandemic.^7^ Before the next pandemic, we have a window of opportunity to improve administrative service data availability and linkage. In this perspective article, we call for collaborative action relevant to policy advisers, parliamentarians, academics, health providers, and early years educators and care providers. We focus on Victoria as one of the states with the most promising opportunities for rapid improvement. Specifically, we (i) consider why data from the first 2000 days are essential; (ii) overview gaps that state‐based MCH service data fill amidst existing Commonwealth and state or territory data assets; (iii) reflect on key lessons from the COVID‐19 pandemic; and (iv) recommend essential steps to drive Victorian MCH service data linkage for informing future policy decisions, service delivery and program evaluation.

## Why do the first 2000 days matter?

The first 2000 days is a critical period for rapid brain development with more than one million neural connections formed each second.^5^ Children's health and development during this period are highly susceptible to the social determinants of child health, an interplay of modifiable societal and environmental factors such as the accessibility of health care, adequacy of household incomes or quality of early education and care.^8^ When disrupted, these social determinants become major drivers of prevalent health problems and avoidable health inequities facing children, including readiness for school, obesity and mental ill health.^8^, ^9^, ^10^ It is now widely understood that early investment in the first 2000 days results in a high rate of return on the reduction of lifetime risk of chronic disease and associated economic impacts.^10^, ^11^ Data from this period are therefore vital for informing decisions on early investment based on the population's needs.

## What data are routinely collected in Victoria within the first 2000 days?

The MCH service dataset is a high value asset. As shown in Box 1, it is the only dataset in Victoria documenting statewide wellbeing of children and parents and developmental milestones in the first 2000 days (preceding school entry). All other available datasets in Victoria document interactions with service systems that flow from health or developmental needs, thus mainly reflecting ill‐health or developmental concerns. MCH service data are routinely collected by Victoria's free, universal MCH service that provides health promotion and intervention for families in the early years.^3^ The most recent MCH service annual report (2017–2018) estimates close to full population coverage for the first home consultation and high retention in the service (over 80% at 12 months, and over 60% at 3.5 years).^12^ According to a 2013 survey, Victoria leads in population coverage compared with other states and territories, although no national MCH database exists for contemporaneous comparisons of MCH data across Australia.^3^

Box 1 **Key datasets in Victoria documenting child health and determinants**

**Table:** 

Domain and topic	State*	MCH	Commonwealth^†^
**Universal care and wellbeing**			
Maternal			
Antenatal care	X	X	
Labour characteristics	X	X	
Maternal mental wellbeing		X	
Neonate			
Birth characteristics	X	X	
Early feeding	X	X	
Newborn screening		X	
Child (0–5 years)			
Physical growth/BMI		X	
Breastfeeding/nutrition		X	
Language		X	
Social/emotional		X	
Gross/fine motor		X	
Immunisations			X
Child (> 5 years)			
Language			X
Social/emotional			X
Gross/fine motor			X
Immunisations			X
**Health determinants and family support**			
Family			
Maternal age	X	X	
Level of English	X	X	
Country of birth/arrival year	X	X	
First‐time mother	X	X	
Marital status	X	X	
Parental employment/education		X	X
Household income/welfare payments			X
Neighbourhood disadvantage	X		X
Housing type			X
Household safety/family violence	X	X	
Child protection	X	X	
**Mortality, morbidity and medications**			
Maternal			
Comorbidities	X		
BMI	X		
Deaths	X		
Hospitalisations	X		
SARS‐CoV‐2 infection	X		
Smoking/alcohol in pregnancy	X	X	
Postnatal depression	X	X	
Family violence	X	X	
Medicare (MBS) ^‡^			X
Medications (PBS)			X
Neonate			
Preterm	X	X	
Stillbirth	X	X	
NICU/SCN admission	X	X	
Child (0–5 + years)			
Deaths	X		
Hospitalisations	X		
Chronic illness	X		
Developmental delay		X	
Medicare (MBS)^‡^			X
Medications (PBS)			X
**Health promotion**			
Family			
SIDS safe sleeping		X	
Breastfeeding		X	
QUIT smoking		X	
Healthy eating		X	
Tooth tips		X	
SunSmart		X	
Communication, language, play		X	
Immunisations		X	
Postnatal depression		X	
Child safety		X	
Injury/poison prevention		X	

BMI = body mass index; MBS = Medicare Benefits Schedule; MCH = maternal and child health service data in Victoria; NICU/SCN = neonatal intensive care unit/special care nursery; PBS = Pharmaceutical Benefits Scheme; SARS‐CoV‐2 = severe acute respiratory syndrome coronavirus 2; SIDS = sudden infant death syndrome. * Centre for Victorian Data Linkage datasets; ^†^ Australian Early Development Census dataset (school‐based tests administered every three years for a subset of the population), Australian Immunisation Register and Person Level Integrated Data Asset datasets; ^‡^ Data are collected for the family rather than the individual.

## The potential for national linkage of data from the first 2000 days

All states and territories have data linkage units, with maternal and child health data linkage being achieved in South Australia, Tasmania, New South Wales and Western Australia.^13^, ^14^, ^15^, ^16^ Given Victoria's high population service coverage and comprehensive data collected, linking MCH service data with other key Commonwealth and state or territory datasets and longitudinal cohorts (Box 1) would create a powerful resource for early intervention policy. Emerging national databases currently lack maternal and child health data, including the Australian Early Development Census, the Australian Child and Youth Wellbeing Atlas, the National Disability Data Asset and the Child Wellbeing Data Asset. Linking Victoria's MCH service data into these databases would be an important step towards more complete integration of MCH data into nationally linked early childhood datasets. This integration would support comparisons of service effectiveness and coverage across Australia and create new opportunities to harmonise and optimise MCH health care provision and outcome monitoring nationally.

## The COVID‐19 pandemic and the first 2000 days: a case study

The COVID‐19 pandemic highlighted the need for timely MCH service data to provide rapid monitoring and reporting of child health and development outcomes.^7^ In Victoria, children faced substantial and immediate transformation of the social determinants of child health, with Melbourne becoming one of the most locked down cities in the world.^17^ In addition, early childcare education and care services closed to most families, and postnatal health services reduced home visits for infants aged over eight weeks, shifting to telehealth and shortening any remaining face‐to‐face appointments to under 15 minutes.^18^

Four years after the onset of the pandemic, we face critical research questions (see Box 2) about the short and long term repercussions of the pandemic on Victoria's COVID‐19 generation. Although a few local cohort studies have used hospital data to report on sick‐child outcomes during the pandemic,^19^ there is a gap in reporting on population‐wide well‐child outcomes such as social and language development, body mass index and infant nutrition. Making routinely collected MCH service data available for linkage could provide an opportunity for answering the urgent questions proposed in Box 2, focusing on evidence that could inform strategies for minimising adverse implications on long term health for the COVID‐19 generation and preparing us for future public health crises.

Box 2 **Population‐based research questions that maternal and child health service data could inform**

- Which coronavirus disease 2019 (COVID‐19) policies advanced children's health?
- Which COVID‐19 policies had negative consequences for children's health?
- How can future policies continue these advances or mitigate these harms during the recovery phase of the COVID‐19 pandemic and in future global crises?

## Rationale for data linkage

The reasons for linking datasets are as follows:

- It can be done. We can look to South Australia's Better Evidence, Better Outcomes, Linked Data (BEBOLD) platform as an example of a well‐child data asset in Australia that is accessible and linkable.^16^ This platform links South Australia's Child and Family Health Services (CaFHS) dataset, similar to Victoria's more comprehensive and higher population uptake MCH service data, with key state or territory and Commonwealth datasets in child protection, hospitalisations, welfare and education. This integration supported reform in South Australia's CaFHS^20^ and has enabled causal evaluation of nurse‐led family home visiting on child development outcomes.^21^
- We have a political window of opportunity. Converging policies and data‐linkage initiatives suggest that it is both timely and urgent to consider systematically integrating Victoria's MCH service data. For example, the national Early Years Strategy 2024‐2034 calls for improved sharing and integration of community and service‐level data to provide a better profile of families and children nationally and locally, measure child health, and report on outcomes.^5^ There is also encouraging progress towards data linkage. This includes the intergovernmental agreement on data sharing in which all Australian governments have committed to sharing public sector data between jurisdictions as a default position and to maximising the value of their data to deliver outstanding policies and services for Australians.^22^ The 2023–2024 federal Budget has also allocated $16.4 million to the Australian Bureau of Statistics to pilot a Life Course Data Initiative.^23^
- The time is now. In the next few years, Victorian MCH services are planning to transition to a new data collection and storage system. Taking into consideration the necessary governance processes, including ensuring enduring Indigenous data sovereignty,^24^ this provides an opportunity to design an MCH service data asset that is similar to high quality National Minimum Data Sets and linkable with other Commonwealth and state or territory data assets. Additionally, MCH linkage would enhance Australia's most recent and largest longitudinal cohort study, GenV, which recruited newborns from 2021–2023 with ongoing early years follow‐up and recruitment of new families.^25^

## Conclusion

It is widely accepted that prevention and early intervention in the first 2000 days of life have lifelong benefits for health and development. Australia has vast data repositories to guide these efforts; however, the only routinely collected, statewide dataset in Victoria documenting wellbeing during these first 2000 days, the MCH service dataset, remains unlinked with other key health and social determinant datasets and longitudinal cohorts. The COVID‐19 pandemic highlighted the need and missed opportunity for accessible MCH data to proactively monitor child health and development. Pandemic responses demonstrated how collectively we can rapidly, proactively and ambitiously shift policy for the betterment of community, no longer asking “what can be done?”, but “what should be done?”. Achieving the potential of the MCH data can be done, we have a political window of opportunity, and the time is now to fulfil our responsibilities both to our communities, from whom we collect the data, and to the wellbeing of our children.

## Competing interests

This perspective article was written as part of Ashleigh Shipton's PhD project “The effect of COVID‐19 pandemic and policies on maternal and child health outcomes in the Western Health region of Victoria: a mixed methods study” funded by the University of Melbourne, Royal Australasian College of Physicians and Murdoch Children's Research Institute. The Royal Children's Hospital Melbourne Human Research Ethics Committee approved Ashleigh Shipton's PhD project as above (reference number: 87751). Melissa Wake was funded by the Australian National Health and Medical Research Council (NHMRC) Principal Research Fellowship 1160906. Sharon Goldfeld was funded by NHMRC Practitioner Fellowship 2026263. Ashleigh Shipton, Meredith O'Connor, Melissa Wake, Sharon Goldfeld, Jatender Mohal and Fiona Mensah's research at the Murdoch Children's Research Institute is supported by the Victorian Government's Operational Infrastructure Support Program. Rhiannon Pilkington was supported by NHMRC CTCS (1187489). Leesa Hooker, Kristina Edvardsson, Helen Lees and Catina Adam's in‐kind support is funded by their teaching and research at La Trobe University. Helen Lees receives in‐kind support from the Municipal Association of Victoria as the maternal and child health policy and program lead. Authors acknowledge that project and salary support for projects unrelated to the present manuscript were received from funding bodies including the NHMRC, Medical Research Future Fund, Paul Ramsay Foundation, State Government of Tasmania, Victorian Government, right@home implementation licence, Australian National Research Organisation for Women's Safety, Victorian Department of Social Services, Family Safety Victoria, Channel 7 Children's Research Foundation, South Australian Department of Human Services, Uniting Communities, Junction Australia, and Commissioner for Aboriginal Children and Young People. Authors acknowledge that payment of honoraria unrelated to the present manuscript were received for lectures, presentations and manuscript peer review. The University of Melbourne had no role in planning or writing this article. The other funders had no role in the planning, writing or publication of this article.

## Provenance

Not commissioned; externally peer reviewed.
